# Considerations for engaging in patient-oriented research with injured workers

**DOI:** 10.3389/frhs.2025.1589643

**Published:** 2025-06-04

**Authors:** Gagan Nagra, Pam Hung, Meaghan Ray Peters, Christine Guptill, Victor E. Ezeugwu, Lynn Cooper, Beverley McKeen, Douglas P. Gross

**Affiliations:** ^1^ Faculty of Rehabilitation Medicine, University of Alberta, Edmonton, AB, Canada; ^2^ School of Rehabilitation Sciences, University of Ottawa, Ottawa, ON, Canada; ^3^ Department of Physical Therapy, University of Alberta, Edmonton, AB, Canada; ^4^ Canadian Injured Workers Alliance, Thunder Bay, ON, Canada; ^5^ Retired, Canadian Injured Workers Alliance (CIWA) Representative, Canadian Union of Public Employees (CUPE), National Occupational Health & Safety Facilitator, Thunder Bay, ON, Canada

**Keywords:** patient oriented research, patient engagement, occupational therapy, knowledge translation (KT), patient participation, workplace, participatory action design (PAR)

## Abstract

**Background:**

Patient-oriented research (POR) incorporates patient-identified priorities and lived experiences into research. Despite their central role in return-to-work (RTW) planning, perspectives and priorities of injured workers are under-represented in Occupational Therapy research. Occupational therapists (OTs) play a key role in RTW research and practice, implementing evidence-based plans and patient-centered care, which positions them well to conduct POR.

**Purpose:**

The purpose of this paper is to identify considerations for POR approaches for OTs to engage injured workers in RTW research.

**Key issues:**

The engagement of injured workers as research partners is not well described or understood in POR. This paper outlines practical considerations for conducting POR with injured workers, addressing challenges such as power imbalances, communication barriers, fears of unemployment, and varying levels of vulnerability. OTs can facilitate knowledge transfer and act as knowledge brokers within the RTW process, leveraging their client-centered practice to lead research that optimally engages injured workers.

**Conclusion:**

Conducting POR with injured workers can shed light on their interactions with health, insurance, and compensation systems. POR approaches can highlight strengths and limitations of available services and systems and promote improved collaboration and knowledge translation and exchange. OTs can apply POR in research and practice to bridge this gap.

## 1 Introduction

### 1.1 Overview

Workplace illnesses and injuries are on the rise with increasing workplace demands, changes to work-life balance, and COVID-19 pandemic-related conditions (1, 2). A worker's recovery and ability to return to work (RTW) is influenced by interactions with multiple service providers (e.g., healthcare workers, case managers), employers, and insurance policies/procedures (3). Injured workers experiencing delayed RTW make up less than 15% of claims but these cases are responsible for 75% of total healthcare and wage replacement costs (4). A main focus has been early RTW to manage claim costs and prevent long-term disabilities (4, 5), yet little input about how to do this has come directly from workers (5).

Patient-oriented research (POR) engages patients and their families and/or caregivers in the research process to focus on the patient care, service providers’ perspectives, and navigating health system policies and procedures (6, 7). In Canada, the objective of POR is for patients, researchers, healthcare providers, and decision-makers to work together in a collaborative manner to establish a healthcare system that is sustainable, accessible, and fair, and ultimately leads to health improvements (23). POR can lead to more effective approaches with higher patient satisfaction through enabling knowledge mobilization to clinical practice; therefore, exploring POR with injured workers may be beneficial for improving rehabilitation and RTW outcomes (23).

The demand for healthcare research that directly addresses patient needs has led to calls for POR, which actively involves patients throughout the research process to ensure relevance in addressing pressing health issues (6, 7). In occupational therapy (OT) research, POR allows clinicians to collaborate closely with people with lived experiences of chronic conditions to enable development of interventions that are both clinically effective and personally meaningful. Given their relationship-focused approach to patient care, OTs are well suited for POR, which aligns with evidence-based, patient-centered care with potential for better outcomes and faster knowledge translation and exchange (KTE) (6, 8). However, OT research has traditionally been clinician-led and missed patients’ perspectives despite its philosophical roots in patient-centeredness. Expanding POR in OT—particularly with injured workers as patient partners to inform RTW research—could foster innovative, impactful insights.

Injured workers lack representation in research despite being the party most impacted by RTW planning. Patient-identified priorities can provide insight to primary causation and thus, solutions to improve care and RTW (23). POR with injured workers should therefore be at the forefront of workplace disability prevention and rehabilitation. Yet, no specific guidelines for clinician-scientists, such as OT researchers, exist to conduct POR with this patient population.

The purpose of this paper is to introduce and apply POR methodology, approaches and frameworks to help engage injured workers in OT research. In addition, perspectives on the need for and barriers to engaging in POR will be examined.

### 1.2 Study design

This manuscript is a commentary paper that utilizes a conceptual and reflective approach to explore the application of POR in OT, particularly in the context of RTW research involving injured workers. Our primary aim was to identify key considerations and offer practical guidance for occupational therapists seeking to implement POR approaches in their research practice.

We grounded our analysis in existing POR frameworks and models, emphasizing their relevance and adaptability to OT. The commentary synthesises insights from the literature on patient engagement and integrates experiential knowledge drawn from clinical practice. Through this dual lens, we examined current limitations and barriers to implementing POR and proposed strategies to address them.

This paper does not report original empirical data but instead provides a perspective designed to support and inspire occupational therapists in conducting more inclusive and responsive research with injured workers. A supplementary material (Supplementary Table 1) provides a step-by-step guide based on the POR framework to assist in the practical application of these principles.

## 2 Why should OTs conduct POR with injured workers?

POR approaches involve people with lived experience to ensure that research focuses on patient-identified priorities, is meaningful to their needs, and ultimately leads to satisfactory and relevant outcomes (7). According to the Canadian Institutes of Health Research (CIHR), POR involves a range of clinical and health research activities, beginning with preliminary human studies, progressing to comparative effectiveness and outcomes research, culminating in the integration of this research into the healthcare system and clinical practice (6). POR approaches can promote integrated KTE, resulting in health systems and clinical applications that have a positive impact on patient care (6). KTE informed by POR necessitates that researchers, healthcare administrators, and clinicians perceive patients as active and valued collaborators in the research process (8).

POR has been conducted with other populations such as individuals living with mental illness, but few POR initiatives have included injured workers. Conducting POR with injured workers can shed light on their interactions with health, insurance and compensation systems and the strengths and limitations of these services and systems (3). Despite best efforts in research, without being informed by the voices and values of injured workers, many findings are difficult for clinicians to integrate into practice and for workers to embrace as patient-centered. OT research utilizing POR can foster sustainable RTW outcomes by aligning RTW programs with workers’ lived experiences, thereby enhancing both effectiveness and durability of RTW initiatives. POR can identify injured workers’ perspectives that can make a positive shift towards developing interventions and systems that are responsive to worker needs. With multiple service providers involved in the RTW process, using POR approaches can not only highlight barriers and limitations in current processes but also provide novel solutions informed by patient-identified priorities and concerns.

At times, insurance policies/procedures present barriers that affect researchers’ abilities to include injured workers as partners, such as focusing only on ‘compensable’ injuries rather than more holistic and client-centered approaches that incorporate psychosocial and contextual aspects (5). Additionally, engagement of injured workers as research partners is not well understood or described in the literature. Guidelines for applying POR in OT research with injured workers have not been developed and should be explored through POR applications with other patient populations (9).

Shaw et al. (10) highlighted that injured workers and other service providers such as claim owners, employers, etc. are typically not included in research to inform best practices in the RTW process. They found that qualitative research is useful to gather opinions and preferences of individuals experiencing work-related health conditions or injury to enhance knowledge about RTW, recovery, and rehabilitation. However, there has been limited research on how to effectively involve and empower injured workers as engaged knowledge brokers. Injured workers may be challenging to engage in POR as patient partners due to barriers such as confidentiality requirements and issues related to compensation and insurance claims. Shaw et al. suggest that more collaboration with RTW service providers is necessary to fully comprehend the role of injured workers as end-users (10). Understanding injured workers’ perspectives and identifying ways to incorporate them through POR may help transform claims processes and RTW research. From an academic standpoint, there are several frameworks, guidelines and resources for conducting POR that we will describe (11).

## 3 Approaches to POR

Several Patient and Public Involvement (PPI) organizations exist to support POR and patient partnership in health research. This includes the CIHR Strategy for Patient-Oriented Research (SPOR) in Canada, Patient-Centered Outcomes Research Institute (PCORI) in the USA and INVOLVE in the UK (12). These organizations have developed policies, standards, and procedures to implement quality and ethical POR.

CIHR created SPOR for researchers to promote patient engagement in health research (6). SPOR was designed for key associates to collaborate and conduct POR in a manner consistent with CIHR principles while keeping patients at the core. The SPOR Patient-Engagement Framework consists of four key principles: inclusiveness, support, mutual respect, and co-build (24). All provinces and territories in Canada have Support for People and Patient-Oriented Research and Trials (SUPPORT) units that implement the SPOR Patient-Engagement framework.

Using Alberta as an example, the Alberta SPOR SUPPORT Unit (AbSPORu) outlines five key steps for researchers to implement when conducting POR. These steps include: Why, Who, How, Engage, and Evaluate. Refer to Supplementary Figure 1 for the AbSPORu steps for patient engagement in health research (24). Patient engagement within the context of POR should be evaluated from the patient partners’ and researcher's point of view (24). For example, the Public and Patient Engagement Evaluation Tool (PPEET) is one resource that can be used to evaluate engagement at various levels of research (11, 25).

Using POR approaches will enable OT researchers to actively involve injured workers as partners, enhancing the relevance and impact of research through their lived experiences.

## 4 Applying POR to engage injured workers in OT research: opportunities and barriers

### 4.1 What has been learned from POR in other populations?

POR has been used with various marginalized groups such as people living with mental illnesses and people living with developmental disorders like autism by adapting the SPOR Patient Engagement (PE) and AbSPORu frameworks (13). These POR approaches can be adapted and applied to OT research with injured workers.

POR studies engaging patients and/or researchers living with mental illness show adaptations to guiding frameworks (13). In Canada, POR recommendations for working with individuals living with a diagnosed mental illness involves avoiding jargon, integrating Priority Setting Partnerships, checking personal and systemic biases, and drawing upon available SPOR SUPPORT units when possible (13). Furthermore, it requires making modifications to the current SPOR framework to include strategies such as providing transparency in objectives and values, addressing stigma, and planning the process carefully based on the needs of the study population (14). Modifications and adaptations can be made throughout the research process to meet the needs of individuals living with mental illness and may include researchers living with mental illness as part of the study team (15).

Autistic people experience social marginalization and may need support to communicate and interact with study teams, which requires additional considerations for POR (26). Current POR guidelines for autism outline considerations for researchers when conducting each step of POR (15, 16). These include providing transparency in processes/procedures, providing literal meaning and concrete examples, avoiding abstract concepts and jargon, and providing options for communication such as tablets, non-verbal communication strategies, etc. (17). The CONtiNuity of carE and support for autistiC adulTs (CONNECT) research project included adults with autism, their caregivers, and multiple service providers using a POR approach (16). It was found that adapting the language used in research to be more suitable to the patient population increased engagement in POR. Adults with autism noted having clear, delineated expectations and clearly defined roles as essential for rapport and conducting POR (16). Another study highlighted the importance of avoiding ableist language and mitigating power imbalances that may arise between the researcher and the patient population (26). Additionally, researchers must ensure they are representing the spectrum when recruiting patients rather than a small representation of the patient population as that can affect generalizability (16). Similar to POR with individuals with mental illness, POR with autistic adults requires adaptations throughout the SPOR steps to facilitate engagement throughout the process.

Due to a lack of resources available to guide OT research to engage injured workers, applying some adaptations from mental illness or autism opens the possibility to use POR methods with injured workers at all steps of the POR process (14). Existing literature from these populations can inform POR in RTW research.

### 4.2 Barriers to POR in RTW research

Incorporating injured workers in every step of a research project is essential for making results reflective of their perspectives and needs. This is consistent with collaborative treatment approaches in OT. Several factors make this challenging, including power imbalances that may be reinforced by the healthcare, insurance and workers’ compensation systems, communication concerns, and job insecurity. Strategies to support injured workers in research as patient partners or participants include outlining their roles and responsibilities on the project, providing flexibility to include their perspectives, and ensuring confidentiality to navigate the complex insurance and workers’ compensation systems as well as workplace social milieus. Adaptations to the AbSPORu and SPOR PE frameworks will be needed to address barriers presented within insurance and workers’ compensation systems and meet the needs of injured workers. A full summary of considerations for conducting research with injured workers using the AbSPORu framework is proposed in Supplementary Table 1.

#### 4.2.1 Prioritize worker perspectives and needs

The first step in conducting POR with injured workers is to consider their perspectives and identified needs. They often feel their opinions and needs are not heard or addressed in RTW planning and their lived experiences, diverse backgrounds and unique contexts are not considered. Using POR approaches and including the perspectives of injured workers will better address the needs of people accessing services (injured workers). However, the SPOR PE framework fails to acknowledge the intricate societal, environmental, economic, and political circumstances that individuals, families, groups, communities, and populations face and how these factors affect health by positioning people and groups within systems of authority and advantage (18).

One way to ensure injured worker perspectives are considered in a holistic and authentic way is to involve injured workers as members of the research team. They may identify gaps in knowledge that other service providers and OT researchers may not view as important. They may also make recommendations that benefit all partners while keeping injured workers’ voices at the forefront. Alternatively, research ideas may be identified with a literature review of previous POR or by conducting interviews with workers in the planning and preparation phase. Sager and James (19), identified four major themes in RTW research from workers’ perspectives including lack of knowledge and understanding of the rehabilitation process, a lack of support for the injured worker, unsatisfying RTW processes, and negative attitudes toward injured workers. Interviewing injured workers to gather ideas or themes to inform research questions or establishing clear roles for workers on the research team can be used to guide research priorities in ways that are meaningful to workers, as has been done with autistic adults (16).

#### 4.2.2 Power imbalances

The most complex barrier to engaging injured workers in POR is likely power imbalances inherent to the RTW environment. There is an intricate web of interdependent hierarchies in the healthcare, insurance, workers’ compensation, and employment systems as well as broader research processes. This impacts how POR is conducted with injured workers. These hierarchical structures, though inherent and a part of various systems, can perpetuate power imbalances between injured workers, healthcare professionals and research team members, preventing progress and collaboration. This is critical to address when conducting POR with injured workers.

Pauly et al. (18) found a lack of acknowledgement of the varying levels of susceptibility in the healthcare system that prioritizes the physician's final say in decision making. This often focuses on healing from injury rather than encouraging overall self-advocacy and wellbeing. This is further supported in the workers’ compensation system where physicians and/or expert consultants on compensation medical boards often have the final say in claims processes with respect to compensable injury. In clinical practice, healthcare professionals (e.g., OTs, physiotherapists) at times make recommendations for care that are not adopted by claim owners, leaving injured workers to perceive that those recommendations were not made. Power imbalances can also exist at the workplace or employer level, healthcare level, and/or claim owner level and can be reproduced in the researcher-patient partner or researcher-participant relationship. In research with injured workers, there is often an unequal dynamic where the decision-making power favors researchers and/or research funders (20). Awareness of these social dynamics is vital to identifying and remedying power imbalances that pervade healthcare and research interactions.

Engaging injured workers in research following the POR philosophy, ‘nothing about me, without me’, can help neutralize power imbalances by strengthening their ability to influence systemic change and counteract the power of claim owners to make decisions (8). It is also important to represent a larger working population when recruiting for POR, including workers with white collar and blue-collar jobs, immigrant workers, seasonal workers, etc. This has been done with autism research in attempts to represent the full autism spectrum (16). This will ensure that a variety of voices within the injured worker population are adequately represented. Specific to injured workers, this includes perspectives from workers with diverse educational levels and socioeconomic backgrounds to provide insight into patient populations that may be underrepresented due to lack of confidence, inadequate resources, and/or communication barriers. To ensure participation of diverse members of the worker population, their needs must be considered during the research planning and preparation phase.

#### 4.2.3 Communication barriers

Communication concerns, such as lack of knowledge and language barriers, can also prevent injured workers from participating in RTW research. Sager & James (19) found that injured workers often feel disempowered from a lack of knowledge and understanding of the rehabilitation process. The ineffective dissemination of information by service providers can lead to confusion and frustration for injured workers. Literature indicates that individuals with lower socioeconomic status (SES) and lower educational levels have higher injury rates (21). Blue collar jobs make up the bulk of injury claims and workers in these industries often have lower SES, lower educational levels, and are often immigrant workers with language challenges. Interpreters may be necessary to address language barriers with patient partners, research participants and/or family members. It is also important to address communication concerns during KTE, ensuring research recommendations are available in various languages and easily understood. This has been done with individuals living with Myocardial Infarction (17). Clearly explaining RTW and insurance/compensation processes and procedures, providing literal meanings and concrete examples, avoiding abstract concepts and jargon, and providing options for communication such as tablets or non-verbal communication, can decrease communication barriers for injured workers and facilitate POR (22).

#### 4.2.4 Fear of unemployment and job insecurity

Barriers related to fear of unemployment and embarrassment among coworkers can negatively impact recruitment and POR engagement, making it difficult for injured workers to discuss their perspectives. Job insecurity is highly prevalent in many labour jobs. SES affects injury risk in complex ways as those facing financial hardship are more likely to take up high-risk jobs, have dangerous working conditions, and are less likely to speak up about working conditions and/or advocate for themselves for fear of losing their job (21). Most work-related injuries impact those with lower SES, lower educational levels, and greater financial hardship, causing increased fear of unemployment (21). Thus, losing time and money to participate in POR may not be a priority for workers as the extra time and expense required in most POR may not result in direct benefits for these individuals. To counteract these concerns, researchers need to ensure anonymity by implementing strict confidentiality procedures that are transparent to participants and appropriately compensate patient partners for their time. Security must also be ensured about information pertaining to intersectionality, power imbalances, and confidentiality. These considerations may facilitate POR by creating a safer space for workers to participate as full patient partners or participants, resulting in greater volume and accuracy of reported experiences and more valid research findings.

Creating safe spaces through rapport building, collaboration, addressing power imbalances, mitigating communication barriers, and providing resources (i.e., time and money to engage) can lead to greater and more representational recruitment of injured workers. Overcoming these barriers to POR will empower all partners in the RTW research process, including injured workers, to inform research-related decisions leading to more impactful and meaningful results.

## 5 Summary of barriers for conducting POR with injured workers

When conducting research with injured workers, it is essential for OT researchers to weigh benefits against risks. We have discussed the challenges and barriers to using POR approaches with injured workers. These include the extra time and expense involved in most POR, and the fact that the research may not result in direct benefits to the workers involved in the research project. One of the most challenging barriers to engaging the injured worker population is the mistrust they often hold with insurance and workers’ compensation systems (22). Injured workers may be concerned about exposing personally identifiable information, which can be risky. Some workers may not engage in POR due to fear of job security as well as fear of consequences within the compensation system due to lack of understanding of processes and procedures. Keeping in mind the SPOR principles of inclusiveness, support, mutual respect, and co-build, while considering the unique challenges and needs of this population, we have described ways that OT researchers can create safer opportunities for injured workers to participate as full research partners.

## 6 Conclusion

Using POR approaches with injured workers is a promising direction for conducting meaningful OT research that improves the health and well-being of workers, improves sustainable RTW, and reduces compensation costs. Collaborating with workers as partners in the research process enables the integration of their unique needs and priorities, ensuring that results are relevant and impactful. OT research using POR can lead to more sustainable RTW outcomes by fostering a sense of ownership and alignment between workers’ lived experiences and the support provided, thus improving both the success and longevity of RTW programs. Although several considerations must be addressed to effectively conduct POR with injured workers, further research is needed to explore how best to implement these approaches and identify optimal ways to involve injured workers in OT research.

### 6.1 Key messages

• The POR framework allows occupational therapy work disability researchers to incorporate the unique views, perspectives, and needs of injured workers, making results more relevant to this population.
• Modifications to the POR framework are necessary to involve injured workers in POR and for their perspectives to be considered.
• This paper outlines practical POR considerations and recommendations for conducting research with injured workers by incorporating their perspectives, addressing power imbalances, improving communication, overcoming fear of unemployment and other levels of varying susceptibility.

## Data Availability

The original contributions presented in the study are included in the article/Supplementary Material, further inquiries can be directed to the corresponding author.
