# Supplementary material for: Considerations for engaging in patient-oriented research with injured workers
Source: Front Health Serv. 2025 Jun 4;5:1589643. doi: 10.3389/frhs.2025.1589643 (PMC12174379; doi:10.3389/frhs.2025.1589643)
Supplement: Supplementary file 1 [file Image1.pdf]

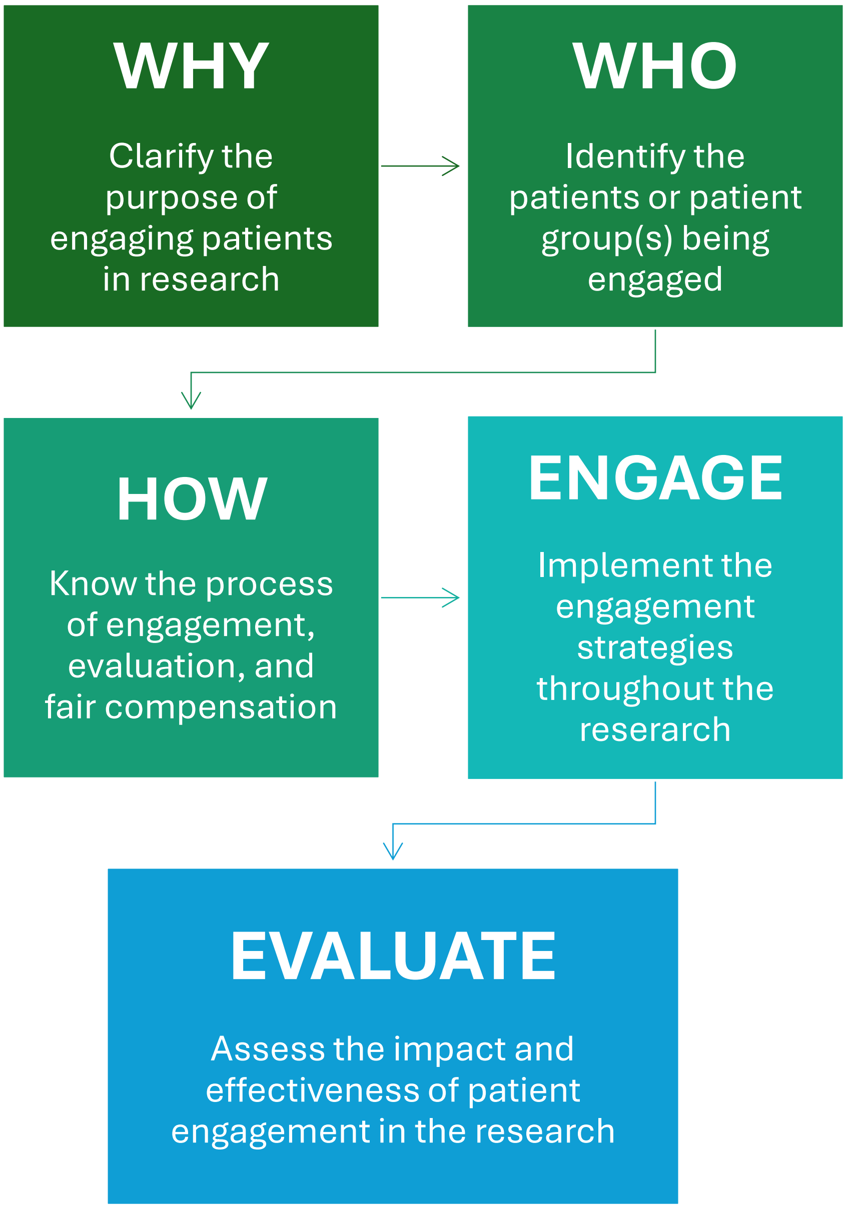


Supplementary Figure 1: Model for POR adapted from Patient Engagement in Health Research: A How-to Guide for Researchers - Alberta Strategy for Patient Oriented Research SUPPORT Unit (AbSPORU). (May 2018). Alberta Strategy for Patient Oriented Research SUPPORT Unit (AbSPORU). https://absporu.ca/resource/patient-engagement-in-health-research-a-how-to-guide-for-researchers/
