# Supplementary material for: Considerations for engaging in patient-oriented research with injured workers
Source: Front Health Serv. 2025 Jun 4;5:1589643. doi: 10.3389/frhs.2025.1589643 (PMC12174379; doi:10.3389/frhs.2025.1589643)
Supplement: Supplementary file 2 [file Table1.docx]

**Table 1:** Considerations for conducting POR with injured workers using the AbSPORu framework

| WHY | ***Why you may want to engage with patients in your research?***   - Injured workers perspectives are underrepresented in RTW research - To gain insight in POR from multiple individual perspectives i.e. Workers’ Compensation Board members - To view how workplaces are affected if we change RTW processes i.e. Employer representatives’ perspectives     ***How to identify what knowledge and perspectives you are seeking from patients?***   - Literature review of injured workers perspectives and gaps in research - Review policies/procedures of Workers’ Compensation Boards - Interviews with injured workers to understand gaps in research for POR     ***What ethical guidelines should be considered when engaging with patients?***   - Confidentiality - Medical vs workplace functional abilities |
| --- | --- |
| WHO | ***Who should you consider engaging in your research?***  Increased diversity of workers’ perspectives in research   - Intersectionality must be considered - Blue collar representation as well as white collar - Immigrant workers - Socioeconomic status - Marginalized population     ***Determine the levels of engagement for patients and researchers***   - Determine with each represented worker what level of engagement they are comfortable with as some may not be able to collaborate and lead while others may only want to learn or consult - Obtain ethics approval - ensure confidentiality at forefront     ***How to recruit patient partners for engagement in research?***   - Consider and address power imbalances - Address language barriers - Address policy/procedures - Involve Clinician-Scientists - Occupational Therapists in recruitment - Use OT models for return-to-work research for engagement and knowledge translation as they view injured workers as patient partners. Collaborative practice is guided. |
| HOW | ***What do you need before engaging patients in research?***    Engaging injured workers at this stage involves addressing recruitment barriers such as:   - Address power imbalances due to fear of employer, unemployment, and/or discrimination in the workplace - Confidentiality parameters and guidelines of how information will be collected and shared - Authorship agreements and guidelines to raise complaints or concerns – this is very important as injured workers may feel fear of unemployment and may be reserved in sharing perspectives   ***A variety of engagement tactics to apply across the research activity cycle must be considered***   - Plan around work if it will affect gradual return to work plans - Plan compensation and reimbursement if necessary (off work may not have enough financial means to be able to be a part of the research) - Transportation if needed - Satisfaction feedback method should be decided before hand for both anonymous and regular feedback   ***How to decide which engagement tactic is right for your research?***   - Knowing what will minimize barriers and maximize participation - Determine participant preferences - Collaborative planning will aide in choosing the correct engagement tactic - Level of engagement will depend on how comfortable the injured worker feels in sharing their perspective - Knowing what resources, you have to be able to provide that engagement strategy i.e. funding, tools, resources, support personnel, legal and policy requirements, ethics, etc.   ***How to budget for patient engagement in health research?***   - Know your participation population (do your research) - Make a budget plan beforehand - Account for rapport building items i.e. refreshments |
| ENGAGE | ***Tips for engagement:***   - Establish clear guidelines - Transparent processes and focus on concrete vs. abstract examples and lay language terms - Foster trust and build rapport and provide safety/security around confidentiality for RTW purposes - Communicate early and regularly - Collaborative perspective and view injured workers’ lived experience equally valuable to available literature - Use plain language and address power imbalances - Communicate based on cultural and linguistic considerations as well as addressing differences in socioeconomic status, lower education, etc. - Encourage space and time wherever possible for feedback or opinions - Address mistrust around Workers’ Compensation Board issues   ***Where to seek support if things go wrong?***   - Acknowledge the problem and actively listen - Ensure support is available for participants and researchers as return to work can be a sensitive topic - Have a mentor for yourself if needed (researchers and team) - Establish a safe communication method prior to starting research for service providers involved |
| EVALUATE | ***How to plan for evaluation?***   - Quantitatively and qualitatively - Use feedback forms/surveys - Use evaluation frameworks (e.g. PPEET document) - Determine what you want to evaluate as the researcher, what was the purpose/aim of POR in this research project? - Did their voices/perspectives get captured? Where themes developed around their needs? - What contextual factors may have prevented authenticity of opinions?   ***Tools to evaluate patient engagement in health research:***   - AbSPORu evaluation guidelines - PPEET tool (3 questionnaires) - CIHR SPOR PE evaluation considerations |

*Note:* This table is guided by 4 key principles of SPOR: Inclusiveness, Support, Mutual Respect, Co-build. Considerations are suggested to the 5 Steps of Patient Engagement for AbSPORu. From *Patient Engagement in Health Research: A How-to Guide for Researchers - Alberta Strategy for Patient Oriented Research SUPPORT Unit (AbSPORU)*. (2022, June 6). Alberta Strategy for Patient Oriented Research SUPPORT Unit (AbSPORU). https://absporu.ca/resource/patient-engagement-in-health-research-a-how-to-guide-for-researchers/
